# Cases of Epilepsy, Compiled from Dr. F. H. Hamilton’s Notes

**Published:** 1858-12

**Authors:** James Mackay

**Affiliations:** Student of Medicine, Buffalo, N. Y.


ART. IV.—*Cases of Epilepsy.* Compiled from Dr. F. H. HAMILTON'S Notes. By JAMES MACKAY, Student of Medicine, Buffalo, N. Y.

CASE I. *Epilepsy produced by Inhaling Carbonic Acid Gas. Recovery.*

Jane Bowden, a servant girl, aged 16. She was healthy at the time of the exposure. She was engaged ironing in a close room, heating her iron by means of a stove filled with charcoal. While she was thus engaged, she suddenly became dizzy and fell to the floor.

For a period of three years after this occurrence, she was subject to epileptic fits; after which time they entirely disappeared.

During her illness she was under the care of Dr. Hamilton, but he does not ascribe her recovery to anything that was done for her, but on the contrary, he believes it to have taken place spontaneously, as far as medical and surgical aid is concerned.

CASE II. In 1855 a young lad, æt. 12, was brought to Dr. Hamilton's office. He was subject to epileptic fits, occasioned by onanism. A cure was effected in a few months by the use of tonics, and by his parents keeping a strict surveillance over him.

CASE III. *Epilepsy from Injury to the Skull. Trephined. Temporary relief.*

Spencer Place, of Kits River, Ogle Co., Ohio, when about nine years of age,fractured his skull by falling from a horse. The wounds closed in about a year, after which the fits commenced.

Oct. 17, 1851, when he was twenty-one years old, he presented himself at the Buffalo Hospital of the Sisters of Charity. He was then suffering from the fits, which occurred every three weeks; at the same time he also complained of pain in the head, usually at the point of former injury.

Dr. Hamilton trephined him before the class. The inner surface of the skull was a little irregular, but not depressed. His recovery was rapid.

He had no fits, or if any, but one or two, from this time until the April following, about six months, at which time they recommenced and occurred at shorter intervals than before. He has not been heard from since.

CASE IV. *Epilepsy from Fracture of the Skull. Continuing five years.*

June 11, 1852. Jeremiah Kery, æt. 36, laborer; he had his skull fractured at the centre of the superior angle of the left parietal bone, five years ago. Four of the pieces were removed; but for five weeks he remained speechless, and palsied on his right side; at the end of that time, eight more pieces were removed by Dr. B., of Sherboygen, Ohio. Very soon after this he recovered his speech and the use of his limbs; but from the time of the accident until now, he has been subject to epileptic fits. Until September last, he used to have these fits every two weeks, but since that time he has stopped drinking, and is now only troubled with them from every two to four months.

He is always warned of their approach for two or three minutes before they take place; he feels a "stagnation" and loss of speech, but he has no pain, nor has he ever any at the seat of injury.

There is an irregular depression at the seat of fracture, and the pulsations of the brain cannot be felt. He will not consent to an operation.

CASE V. *Epilepsy produced by the Firing of a Cannon, and by speaking in a Crowded Room. Continued eleven years.*

Sept. 3d, 1855. R. R. Merritt, æt. 39 years, was a trunk maker, and had always been healthy until the night of the 3d of July, eleven years ago, when he made a speech at 10 o'clock in the evening, in a crowded room, on the occasion of a public school exhibition, at S., Chemung county. The room was very much crowded and very warm. His speech occupied about fifteen minutes in its delivery. He went to bed about 12 o'clock, midnight, and at 2, A. M., got up and assisted in firing off a small cannon, (it was a musket barrel.) The cannon was only fired once. He was well when he got up, and he does not think that the discharge of the cannon affected him. He had a fit a few moments after the cannon was fired, it occurring while his brother was in the act of pouring powder into his hand.

He has had about twenty-five fits every year for the last three or four years, and before that time he had about twelve a year. He was without a fit for six months at one time, (after a "low billious" fever.) He does not remember ever having more than two or three fits, at any hour except before breakfast. He had a fit six days ago, and for the last three days he has had occasional vertigo.

Case VI. *Epilepsy produced by Pregnancy. Continuing fourteen months. Cured by Weaning Child.*

March 28, 1856. Mrs. M. S., æt. 24, consulted Dr. Hamilton, after having been married about sixteen months. Soon after she had become *enceinte,* she had an epileptic fit; she also had several others during her pregnancy, and three since her delivery. She is nursing her child, but does not menstruate. Her fits generally take place at night, and while she is in bed.

About five months after her marriage, her right elbow began to swell, and it has since continued to increase in size until the joint has become completely anchylosed.

Dr. Hamilton believed all these consequences to be due to an arrest of the natural functions of the uterus; and he advised her to wean the child as soon as she could, and not to become pregnant again for some time.

His advice was followed, and in about five months she was completely cured of her fits, and the elbow joint was restored to its natural condition.

Case VII. *Epilepsy from a Fracture of the Skull.*

Leo Keogh, æt. 26, was struck with a heavy block, Sept. 12, 1855. His skull was extensively fractured, and the dura mater wounded.

Dr. Hamilton operated, removing about three square inches of the skull. This operation was followed be hernia cerebri.

In February, 1857, Dr. Hamilton found a fistulous opening still existing on the scalp. The right arm and leg were at the same time partially paralyzed. He had also epileptic fits.

Dr. H. removed a small piece of bone from the opening on the scalp, and the wound healed up.

From Dr. Hamilton's Fracture Tables, published in 1853, with a supplementary table on "Fractures of the Skull," we draw also the following facts:

Case VIII. *Epilepsy from a Fracture of the Skull. Increased by marriage; terminating in Idiocy and Death. (Case 144 of tables.)*

S. H., æt. 9 years. Had fracture of the parietal bone near its middle. He was married when he was twenty-three years old, and soon after he began to have epileptic fits; these fits continued until he became idiotic, and he died when he was thirty years of age.

Case IX. *Epilepsy from a Fracture of the Os Parietal of the left side when two years old. Continued four years. Not cured.*

W. M. H. This boy was kicked by a horse. The bones were immediately removed; but the wound *has never closed.* When three years old he had hemiplegia of *right side.* Has had nervous pains from that time to the present, commencing in right hand and extending to head. At seventeen he had epilepsy, which has continued until now. The pulsations of the brain can be felt distinctly.

Case X. *Epilepsy from Fracture of the Skull. Death on the thirty-sixth day. (Case 161 of Tables.)*

H. V., æt. 3. Fell three feet upon a stove, which produced a wound on the scalp in the forehead. Epilepsy set in in a few weeks.

The child did not seem to be seriously injured, until the thirty-fourth or thirty-fifth day after the accident. He then became gradually comatose. The wound had never healed, but there was a slight swelling and discharge still continuing.

On the thirty-sixth day his coma was complete, but no paralysis. Other means having failed, he was trephined by Dr. Hamilton, at the seat of injury. The outer plate was broken and loose; the inner not broken. No pus. The dura mater was not penetrated. The patient was not relieved, and died on the thirty-sixth day after the injury.

The autopsy disclosed no pus within the skull.

Note. For a case of epilepsy treated by circumcision of the cicatrix, by Dr. Hamilton, see this Journal, vol. v, p. 460.

